# Formalizing Lacanian psychoanalysis through the free energy principle

**DOI:** 10.3389/fpsyg.2025.1574650

**Published:** 2025-06-06

**Authors:** Lingyu Li, Chunbo Li

**Affiliations:** ^1^Shanghai Mental Health Center, Shanghai Jiao Tong University School of Medicine, Shanghai, China; ^2^Shanghai Key Laboratory of Psychotic Disorders, Shanghai Mental Health Center, Shanghai Jiao Tong University School of Medicine, Shanghai, China; ^3^School of Psychology, Shanghai Jiao Tong University, Shanghai, China

**Keywords:** active inference, free energy principle, Lacan, psychoanalysis, neuropsychoanalysis

## Abstract

This study presents a computational formalization of Lacanian psychoanalysis using the framework of the free energy principle (FEP)—a theoretical framework for modeling self-organizing systems across multiple scales. We first examine the theoretical compatibility between the two frameworks, highlighting their shared (1) Kantian epistemological foundations regarding the unknowability of reality, (2) constructive nature of internal representational systems, (3) non-linear temporal dynamics that combine prediction and retrospection, and (4) emphasis on representation failures as key driving forces. Building on these convergences, we develop a computational framework that implements core Lacanian concepts through the FEP framework. Through multi-level simulations, this framework captures the interdependence of *three orders* as a message-passing network, formalizes *desire* as generalized synchronization between subjects' Symbolic orders, and models *the Other* through collective dynamics. This FEP-based reformulation renders traditionally obscure Lacanian concepts computationally tractable, thereby establishing a conceptual bridge between Lacanian psychoanalytic insights and cognitive science.

## 1 Introduction

Jacques Lacan, drawing from psychoanalysis, philosophy, and anthropology, developed a sophisticated portrayal of human subjectivity across bodily, interpersonal, and social dimensions (Homer, 2004). His theoretical contributions, known as Lacanian psychoanalysis, have generated unique insights into mental disorders (Mills and Downing, 2018; De Waelhens and Ver Eecke, 2001), identity (Bailly, 2023), cognitive linguistics (Bazan et al., 2021), and artificial intelligence (Possati, 2020). While highly influential in the humanities, Lacan's work has faced persistent criticism for its obscure terminology and often intentional confusions, frequently dismissed as “fashionable nonsense” in scientific discourse (Sokal and Bricmont, 1999). Recent advances in cognitive science, particularly the free energy principle (FEP) (Friston, 2010), provide formal tools to bridge this divide. This study, therefore, presents an exploration of reformulating core Lacanian concepts into computationally tractable forms that can be simulated and tested.

We employ FEP as the mathematical framework for our formalization not only because of its broad applicability across cognitive systems but, more importantly, due to the fundamental convergence between FEP and Lacanian psychoanalysis. FEP has successfully unified our understanding of self-organizing systems across multiple scales, from neuronal dynamics (Isomura et al., 2023), self-consciousness (Limanowski and Blankenburg, 2013), action, perception, and learning (Friston, 2010), to communications (Friston and Frith, 2015) and collective behaviors (Heins et al., 2024). Through careful examination, we identify deep theoretical alignments between these two frameworks. Both emphasize that one must leverage one's internal representational systems to actively infer and construct beliefs about the external world, of which precise states are always concealed. Moreover, these representational processes are characterized by a non-linear temporal structure and are driven by unrepresented elements in both frameworks. This convergence suggests that more than a formal language; it offers a natural embodiment of Lacanian theory.

We implemented a series of computational simulations to formalize core Lacanian concepts, including three orders (Real, Symbolic, and Imaginary; RSI), the Borromean knot, desire, the formula of fantasy, and the Other. At the individual level, we proposed an FEP-RSI model building on neuropsychoanalytic mappings of the three orders onto brain regions (Dall'Aglio, 2019), reproducing the interdependent nature of RSI through message-passing. In dyadic simulations, desire is modeled as generalized synchronization between subjects' Symbolic orders, offering a formal account of Lacan's metaphoric mechanism of desire. The triadic simulations interpret the Other as collective dynamics that emerge from complex desire relationships. Besides the formalization of concepts, our simulations embodied several key Lacanian conclusions in a computationally intuitive way as well, such as “man's desire is the desire of the Other,” and “the Other does not exist” (Lacan, 2001). The implementation is accomplished with Python (Heins et al., 2022) and the original code is available on GitHub.^1^ Through these computational simulations, we demonstrate that Lacanian psychoanalysis can be rigorously formalized within the mathematical framework of FEP while preserving the theoretical depth and clinical insights of Lacanian theory. This formal bridge potentially fosters mutually beneficial dialogues between these two approaches to understanding the human mind.

## 2 Theoretical background

### 2.1 Lacanian psychoanalysis

Lacanian psychoanalysis stems from a radical re-reading of Freudian theory, emphasizing the role of language and social structures in constituting human subjectivity. Central to his theoretical framework is the basic classification system of subjects' psychoanalytic experiences: the three *orders*—the **R**eal, the **S**ymbolic, and the **I**maginary (RSI) (Evans, 2006). These three orders are interconnected through the topological structure of the Borromean knot, where the removal of any single ring leads to the collapse of the entire structure (Thurston, 2018).

Specifically, the Imaginary order relates to how we engage with our environment through immediate sensory experiences. The term “imaginary” emphasizes that these seemingly direct experiences are fundamentally shaped by subjective perspectives, making them illusory by nature. This idea aligns with the cognitive science perspective that perception is a process of belief formation, i.e., “perceiving is believing” (Fletcher and Frith, 2009).

Unlike immediate sensory experiences, the Symbolic order enables abstract thinking and communication between individuals through the shared systems of society, such as language, social rules, and cultural systems. To be acknowledged by society, subjects must internalize these pre-existing systems. This internalization process structurally transforms the subject's understanding of the world and themselves by embedding a third party into subjectivity. The concept of *the Other* (with a capital O) describes this transcendent third party while emphasizing its externality: it exists beyond any individual but shapes everyone's thoughts and behaviors (Lacan, 2001). While the Other is not a physical entity, subjects often treat specific roles as its representatives, such as parents and authority figures, who influence subjects' thoughts and behaviors by occupying the position of the Other. The internalization of these concrete representatives constitutes the manifestation of one's *desire*.

The Imaginary and Symbolic orders provide two distinct ways of representing the environment through which subjects construct their belief systems about the world, i.e., *psychic reality*. These representational systems operate within a temporal structure of *logical time*. Instead of a linear flow from past to future, logical time is a structure where “the past anticipates a future within which it can retroactively find a place” (Hook et al., 2022). This means that past experiences shape how we anticipate future events, while future events can retrospectively change the meaning we attribute to past experiences.

The Real designates what lies beyond the representations of the Imaginary and Symbolic orders—a “being-in-itself” (Feldstein. et al., 1994). This non-representational feature manifests in two aspects: first, it refers to a biological substrate of human existence—the psychical body's autonomous functions, such as primary emotion, immune processes, and cardiovascular activities (Evans, 2006). Second, similar to Kant's concept of *thing-in-itself*, it points to an inherent unknowability of the precise environment, or *material reality*. In other words, there is always a gap between psychic reality and material reality.

Due to this fundamental gap, the subject continuously encounters failures during its interactions with the environment. These failures drive the subject's incessant attempts to represent what resists representation; this compulsive tendency is called *repetition*. And the item that remains unrepresented during repetition is named as *object petit a*. The object petit a thus represents the very gap between psychic reality and material reality. It is the cause of failures that structure desire as a repetitive effort to eliminate this gap (Lacan, 2001).

In summary, Lacan articulates a model of subjectivity in which the subject uses their internal representational systems, operating within a non-linear temporal structure, to continuously attempt to grasp the external environment that fundamentally resists complete representation.

### 2.2 Free energy principle

To survive, living systems must interact with the environment, including perceiving and changing it. The Free energy principle (FEP) provides a universal principle for understanding these interactions between the environment and organisms across multiple scales, ranging from neurons and brain regions to consciousness and both individual and collective behavior (Isomura et al., 2023; Limanowski and Blankenburg, 2013; Heins et al., 2024; Solms, 2019).

Aligning with Kant's notion that we can only perceive phenomena and not the thing-in-itself, the starting point of FEP is that the precise state of the environment is usually concealed (referred to as the *hidden state*), and living systems can only infer this hidden state from observations by leveraging their *internal model*s (Hohwy, 2018). From this perspective, perception is considered an “unconscious” inference about which hidden state causes the current sensations (Parr et al., 2022). This inferential process follows Bayes theorem:


$$P\left(s|o\right)=\frac{P\left(o|s\right)P\left(s\right)}{P\left(o\right)}$$

where *P*(*s*|*o*) denotes the posterior probability of the hidden state *s* given observation *o*, *P*(*o*|*s*) is the likelihood of observation *o* given state *s*, *P*(*s*) stands for the prior belief about state *s*, and *P*(*o*) represents the marginal likelihood of observation *o*. This equation formalizes how organisms update their beliefs about hidden states by combining prior expectations with new sensory evidence. However, exact Bayesian inference is usually computationally intractable as it requires marginalizing over all possible hidden states.

**Figure 1:**
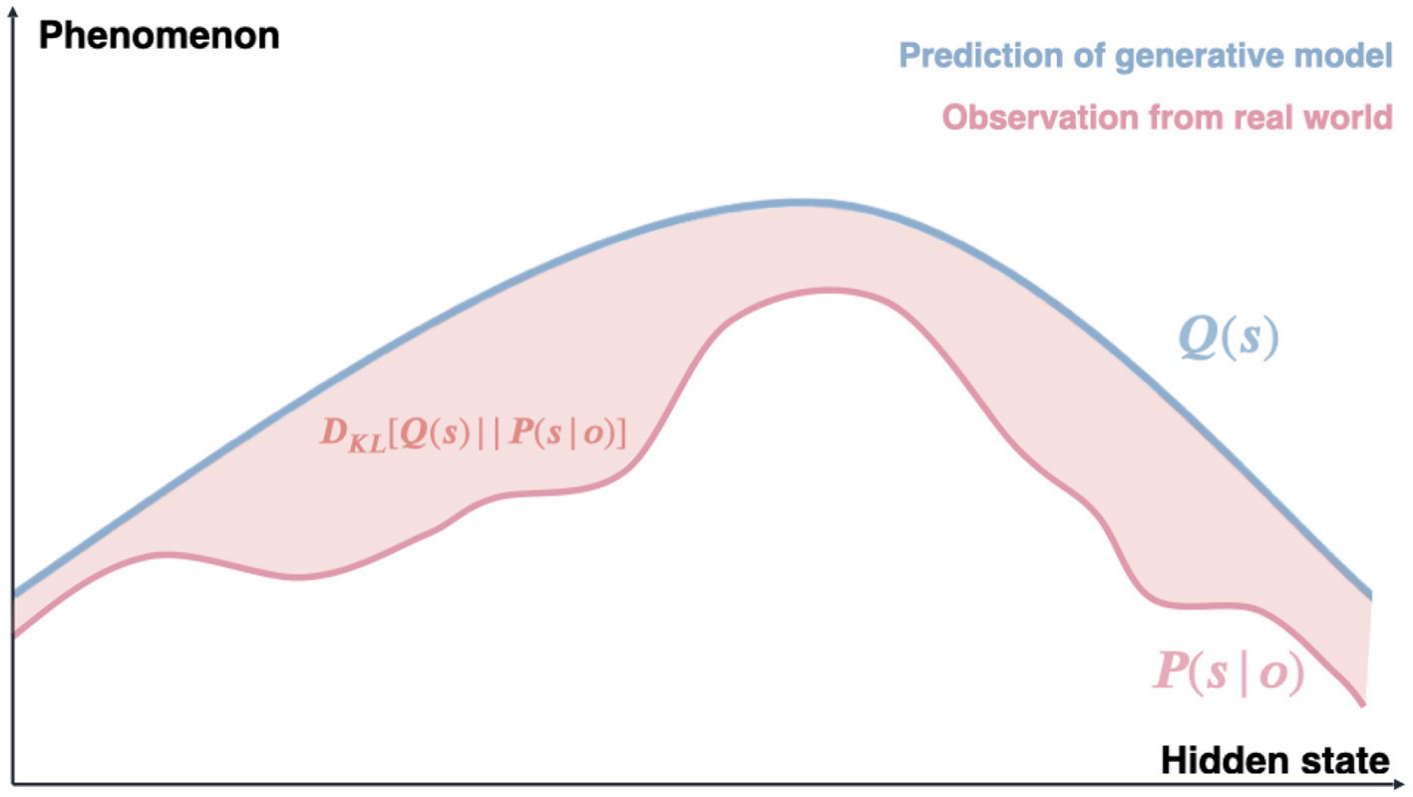
Variational inference as an approximate Bayesian inference. The blue line Q(s) represents the prediction distribution generated by the internal model, while the red line P(s|o) shows the precise posterior distribution derived from real-world observations. The shaded area between these distributions represents the Kullback-Leibler (KL) divergence, which quantifies the difference between the approximate and true distributions. Minimizing this KL divergence through variational inference allows the system to find the best approximation of the true posterior, balancing model complexity with accuracy in explaining sensory data. This process formalizes how organisms update their beliefs about hidden states by combining prior expectations with new sensory evidence.

In biological systems, this inference is accomplished through interactions between top-down predictions based on priors and bottom-up sensations mediated by likelihood, rather than passively receiving environmental stimuli (Fletcher and Frith, 2009). The goal is to achieve optimal convergence between the inferred hidden state *s* generated by internal models and the true hidden state s^*^ of the external world. Consequently, perception (as subjective inference) is not necessarily accurate, which helps explain illusions as instances of inaccurate inferences (Parr et al., 2022). This inferential process is formalized as *variational inference*, an approximate Bayesian inference method (Figure 1) (Blei et al., 2017). Variational inference is an optimization problem that aims to find the best approximation (inference):


$$q^{*}\left(s\right)=\arg\underset{q\in Q}{\min}\text{KL}\left(q\left(s\right)∥p\left(s|o\right)\right)$$

where *KL* is the Kullback-Leibler divergence between two distributions, expanded as:


KL(q(s)‖p(s|o)=𝔼[logq(s)]−𝔼[logp(s,o)]+logp(o)

Because log *p*(*o*) is an intractable constant, the optimization uses an alternative term—evidence lower bound(ELBO):


(1) $$\begin{array}{l} \text{ELBO}\left(q\right)=𝔼\left[\log p\left(s,o\right)\right]-𝔼\left[\log q\left(s\right)\right] \end{array}$$

(2) $$\begin{array}{l} \;=𝔼\left[\log p\left(s\right)\right]+𝔼\left[\log p\left(o|s\right)\right]-𝔼\left[\log q\left(s\right)\right] \end{array}$$

(3) $$\begin{array}{l} \;=𝔼\left[\log p\left(o|s\right)\right]-\text{KL}\left(q\left(s\right)∥p\left(s\right)\right) \end{array}$$

In the context of FEP, variational free energy (F) is equal to the negative ELBO (i.e., maximization of ELBO is equivalent to minimization of variational free energy):


F=KL(q(s)|p(s))-𝔼[logp(o|s)]

The internal model with minimal variational free energy represents the best explanation for sensory data that balances the model's complexity (the KL term) and accuracy (the expected likelihood). The KL term, which quantifies the dissimilarity between priors and posteriors, is also known as *Bayesian surprise* and indicates the magnitude of necessary Bayesian *belief updating*. In addition to updating beliefs, living systems can take actions to collect new evidence or change the environment to reduce complexity or increase accuracy in the future. Consequently, decision-making and planning also follow this minimization principle, but in an expected (predictive) manner. For conceivable actions π, the *expected free energy* (G) is estimated:


(4) $$\begin{array}{l} \text{G}\left(\pi \right)=\text{KL}\left(q\left(\tilde{s}|\pi \right)|p\left(\tilde{s}|C\right)\right)-{𝔼}_{q\left(\tilde{s},\tilde{o}|\pi \right)}\left[\log p\left(\tilde{o}|\tilde{s},\pi \right)\right] \end{array}$$

where *C* includes preferences, and the symbol ˜ denotes expected inferred states and observations. The policy with minimal expected free energy balances the complexity and accuracy of future states. This optimal policy is interpreted as a “belief that one will minimize free energy in the future,” thus conceptualizing actions themselves as a form of inference (Parr et al., 2022).

FEP posits that living systems in environments with hidden states continuously perform Bayesian inference using internal models that combine prior beliefs with sensory evidence to approximate true environmental states while also striving to minimize future expected uncertainty.

### 2.3 Theoretical convergence

The introduction of FEP and Lacanian theory reveals their deep conceptual alignment. First, both frameworks are grounded in the Kantian epistemological foundation of the thing-in-itself: the inherent impossibility of direct access to reality. This fundamental limitation manifests as the concept of *hidden state* in FEP and *material reality* in Lacanian theory. The shared premise leads to a similar understanding of how subjects relate to the external world. Both theories propose that our experience of the external world is necessarily constructed through subjective processes—what FEP terms as *perception-as-inference* and what Lacan describes through *the Imaginary*. As Sass (2015) notes, this implies a world that is “created by humans rather than found.” These constructive processes operate through the subject's internal representational systems, establishing a duality: in FEP, between internal models and hidden states; in Lacanian terms, between psychic reality and material reality.

Another parallel lies in their conceptualization of time. Both theories emphasize a non-linear temporal structure that combines prediction and retrospection. This alignment is explicitly evident in their respective descriptions:

“variational free energy has a retrospective aspect [...] (minimal expected free energy is the) belief that one will minimize free energy in the future” (Parr et al., 2022).

“the past anticipates a future within which it can retroactively find a place” (Hook et al., 2022).

In FEP, the subject forms predictions about future events based on prior beliefs, which are subsequently validated or revised as new sensory evidence becomes available. Similarly, in Lacanian theory, while past experiences shape the subject's anticipation of the future, subsequent events can retrospectively alter the meaning attributed to those past experiences. This dynamic temporal interplay has been acknowledged as a crucial mechanism underlying various cognitive phenomena, including real-time perception in neural systems (Hogendoorn, 2022), sense of agency (Riemer, 2018), experiences of free will (Kühn and Brass, 2009), and counterfactual inference (Miyamoto et al., 2023).

As subjects' representational systems engage with the external world through this non-linear temporal structure, the essential duality inherent in both frameworks necessitates an inevitable representational gap. In FEP, this gap manifests as *free energy*- the divergence between internal models and the hidden states of the external world - which motivates systems to continuously update their beliefs and initiate actions to minimize this discrepancy. In Lacanian theory, this same structural gap appears as *object petit a*—the irreducible remainder between psychic and material reality—which functions as the fundamental driver of subjects' desire.

These profound structural parallels between FEP and Lacanian theory indicate that FEP serves not merely as a mathematical language for modeling Lacanian psychoanalysis. Instead, it constitutes a computational framework that naturally embodies Lacanian concepts, enabling core psychoanalytic insights to be expressed in rigorous mathematical terms and implemented via concrete computational processes.

## 3 Materials and methods

### 3.1 Neural basis of RSI

Building on the neuropsychoanalytic framework proposed by Dall'Aglio (2019), we map Lacan's three orders onto distinct brain networks to establish a neurobiological foundation for our computational model. This mapping bridges Lacanian abstract concepts with neural implementations, serving as an intuitive framework rather than claiming precise anatomical boundaries. In Table 1, we provide a detailed mapping that elaborates on the neural domains, function examples, and relevant FEP studies.

**Table 1:** A neuropsychoanalytic mapping of Lacanian three orders to neural domains.

**Order**	**Neural domain**	**Functions**	**Related FEP research**
Real	Upper brainstem & Diencephalic system	Interoception	Paulus et al., 2019
		Emotional awareness	Smith et al., 2019
		Consciousness	Vilas et al., 2022
		Physiology	Sedley et al., 2024
Symbolic	Prefrontal- Parietal network	Communication	Friston and Frith, 2015
		Self-esteem	Albarracin et al., 2024
		Culture	Kastel and Hesp, 2021
		Understanding	Parr and Pezzulo, 2021
Imaginary	Parietal- Occipital network	Body image	Tremblay et al., 2021
		Intentional actions	Priorelli and Stoianov, 2023
		Self-other distinction	Lanillos et al., 2020
		Theory of mind	Hipólito and van Es, 2022

The Real maps onto the upper brainstem and diencephalic system, which regulate primary affect, homeostatic functions, basic drives (hunger, thirst), and autonomic responses, representing the biological substrate of human existence. The Symbolic links to the prefrontal-parietal network, involving language processing, abstract reasoning, and sociocultural interactions, corresponding to linguistic and cultural representations. The Imaginary associates with the parietal-occipital network, responsible for visual processing, spatial cognition, body schema representation, and motor planning and control, reflecting immediate experiential aspects.

Research demonstrates that functions across these neural networks operate according to FEP, as evidenced by numerous modeling studies (see Table 1). Importantly, this universality of FEP across different modalities enables us to treat these networks as computationally equivalent entities, each implementing free energy minimization within their respective domains.

### 3.2 FEP-RSI model

**Figure 2:**
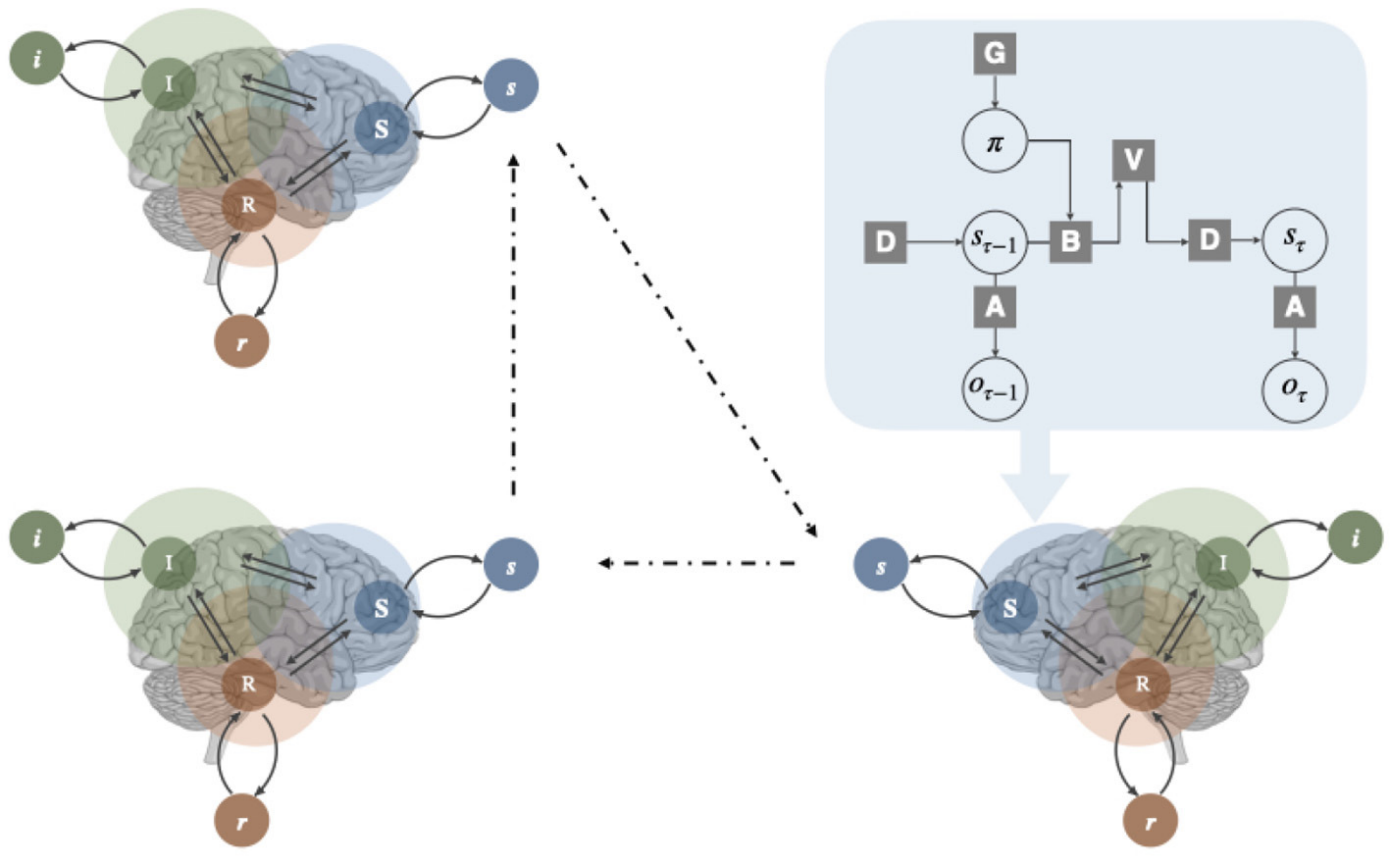
The FEP-RSI model and its implementation in individual and collective dynamics. **Upper-left**: illustration of FEP-RSI model showing the Real (R), Symbolic (S), and Imaginary (I) orders mapped onto corresponding brain networks. **Upper right**: the architecture of a single FEP unit. Collective dynamics between multiple agents are implemented through synchronization of their Symbolic orders, enabling the emergence of interpersonal desire and the Other.

Building on our neuropsychoanalytic mapping framework, we designed the FEP-RSI model to capture the dynamics of Lacan's three orders within the FEP framework (Figure 2 upper right). While the previous section established correspondences between RSI and specific neural networks, our model deliberately abstracts away from simulating these neural functions directly. Instead, we focus on capturing the essential dynamics of RSI interactions. This level of abstraction enables a computational investigation of key Lacanian concepts while limiting the complexity of modeling specific neurobiological processes.

Considering the computational equivalence of the three orders, we implement each order as an FEP unit operating in respective discrete state space *S*. As illustrated in Figure 2 upper left, each unit maintains its own internal model that comprises a likelihood matrix **A** that maps hidden states to observations; a transition matrix **B** that defines state transitions under different actions; a preference vector *C* representing desired observations; and a prior belief vector *D* over hidden states.

At each time step τ, each unit operates through three core mechanisms:

(1) State inference through approximated posteriors:


$$q\left(s_{\tau }\right)=\sigma \left(\log{\text{A}}_{o_{\tau }}+\log D_{\tau }\right)$$

where σ denotes the softmax function, **A**_*o*_τ__ is the likelihood of the current observation *o*_τ_, *D*_τ_ represents the prior belief, and the posterior *q*(*s*_τ_) becomes the prior belief for the next time step τ + 1.

(2) Action planning using expected free energy:


$$G\left(\pi \right)=H\left(\text{A}\right)q\left(s_{t}|\pi \right)+KL\left(q\left(o_{t}|\pi \right)∥C\right)$$

where *H*(**A**) is the entropy of the likelihood matrix, while *q*(*s*_*t*_|π) and *q*(*o*_*t*_|π) are expected states and observations under three possible policies π ∈ {*s* + 1, *s* − 1, *s*}.

(3) Message passing through prediction errors quantified by variational free energy:


$$F=\text{KL}\left(q\left(s_{\tau }\right)∥{\text{A}}_{o_{\tau }}\right)-\log P\left(o_{\tau }\right)$$

which is equivalent to the equation we introduced in Section 2.2. Here, the variational free energy quantifies the total amount of prediction errors. The equivalence arises because both terms in *F* represent different aspects of prediction error: the KL divergence measures the error in state estimation, while the log evidence term captures the error in sensory prediction. Minimizing variational free energy corresponds to maximizing Bayesian model evidence—a process of self-evidencing (Hohwy, 2016) – while simultaneously minimizing prediction errors.

Overall, the model implements each order as an autonomous FEP unit while enabling interactions between units through message passing, thus maintaining both the independence and interdependence characteristic of RSI. Notably, the FEP unit for the Real specifically models its biological and affective dimension—the first facet of the Real as discussed in Section 2.1. The second facet of the Real, the fundamental gap between psychic reality and material reality, is represented by the variational free energy that the entire system seeks to minimize, thereby driving its dynamics.

### 3.3 Implementation

We implemented three levels of simulations to investigate key Lacanian concepts using the FEP-RSI model: individual dynamics demonstrating the Borromean knot structure, dyadic synchronization formalizing desire, and triadic collective behaviors manifesting the Other. All simulations were conducted using Python 3.10 and the Pymdp package (Heins et al., 2022), with the source code available on GitHub (see footnote 1). Each simulation ran for 15 time steps and was visualized through 3-D trajectory plotting to capture the dynamics of the FEP-RSI model, providing an intuitive representation of psychic reality.

First, we simulated the individual dynamics using varying message-passing weights to illustrate the interdependence of RSI. To examine this interdependence, we introduced a perturbation in the Symbolic by initializing the hidden state to 1 while setting its preference to 4. We then observed how this perturbation propagated through the RSI structure under different weights, ranging from zero (no coupling across RSI) to 2.0 (strong coupling).

To investigate desire as generalized synchronization (Friston and Frith, 2015), we implemented a dyadic simulation with two agents that possess identical internal FEP-RSI models but distinct initial states. In line with Lacan's conceptualization of desire as a metaphor (Fink, 2017), we designed each agent to infer the other's Symbolic state as its own preference, thereby modeling desire as an attempt to synchronize internal models through the Symbolic order.

Finally, to examine the emergent property of the Other, we extended the model to a triadic configuration with three FEP-RSI agents arranged in a triangular desire structure (A → B → C → A). This triangular arrangement constitutes a minimal group capable of manifesting the complex dynamics of collective desire, wherein each subject's desire is necessarily mediated through the other participants. Using this configuration, we investigated how collective dynamics emerge from interacting desire processes operating within a social network structure.

## 4 Results

### 4.1 Individual dynamics: RSI as a message-passing network

To validate that our FEP-RSI model captures the interdependent nature of Lacan's three orders, we first investigated the dynamics of a single subject when the Symbolic order is perturbed. Lacan originally illustrated this interdependence using the Borromean knot topology (Thurston, 2018). This topological concept emphasizes that the three orders function as an integrated system, where perturbations in one order necessarily propagate to others through their mutual dependencies, all organized around object petit a (Evans, 2006). In our implementation, we modeled this interconnected structure through message passing of prediction errors among the three orders, with connection strengths parameterized by precision weights.

**Figure 3:**
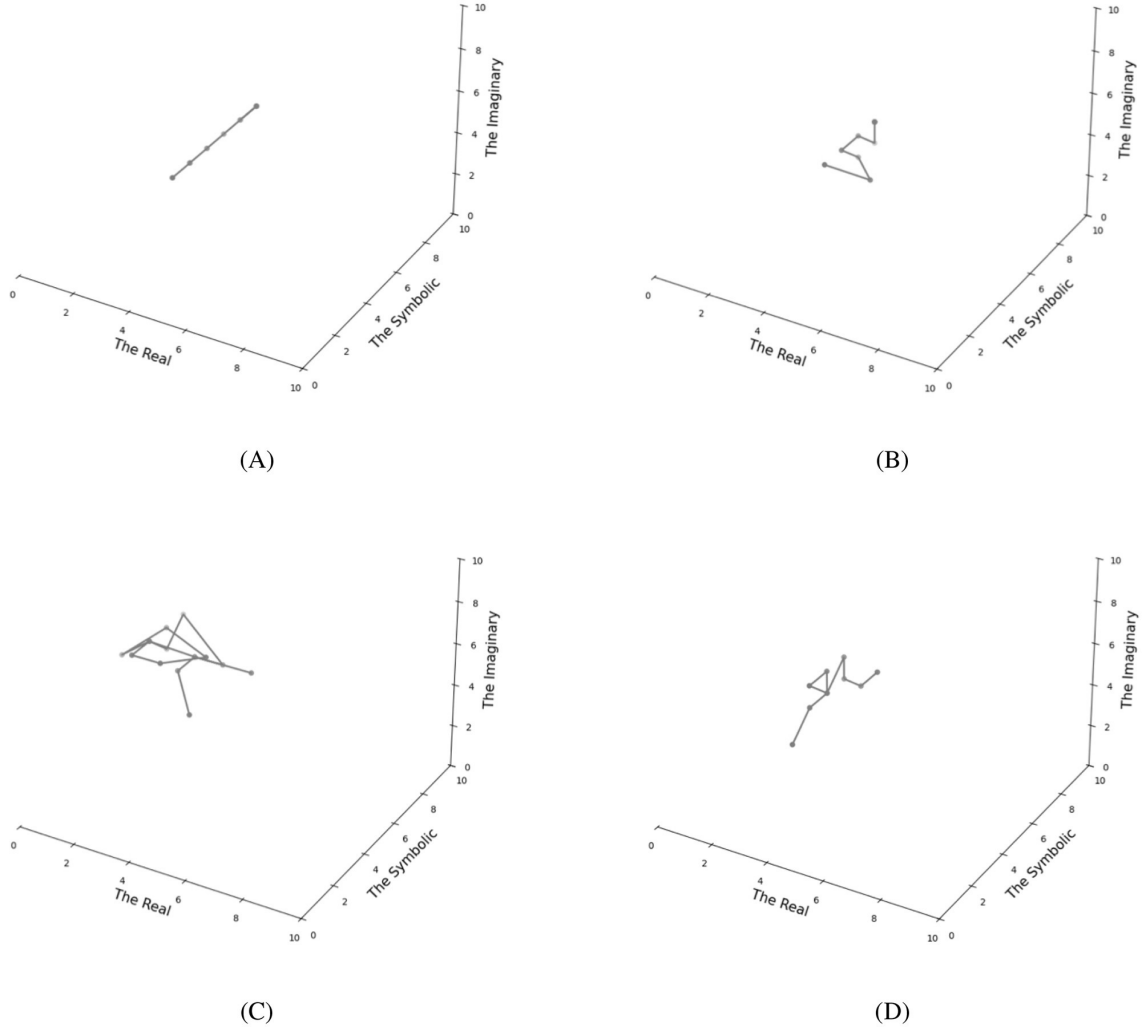
Individual FEP-RSI model dynamics under different message passing weights, demonstrating the Borromean knot structure of RSI. **(A)** Zero-weight condition (all weights = 0) showing independent evolution of the three orders with no interaction between them. **(B)** Moderate uniform precision weights (all weights = 1.5) showing coupled dynamics where perturbation in the Symbolic order propagates to other orders. **(C)** High uniform precision weights (all weights = 2.0) demonstrating stronger inter-order coupling. **(D)** Asymmetric weight configuration (Real = 1.5, Symbolic = 2.0, Imaginary = 1.0). Each trajectory plot shows the evolution of states across the Real, Symbolic, and Imaginary dimensions over 15 time steps.

Figure 3 illustrates the trajectories of RSI states under varying message-passing weights. To establish a baseline, we began with a hypothetical condition where all three weights were set to zero (Figure 3A). This resulted in the independent evolution of the three orders with minimal interaction—a condition that, while theoretically informative, contradicts both neural organization principles and Lacanian theory. We then implemented the message-passing mechanism with increasing weight values to model RSI interdependence. At moderate precision levels (1.5, Figure 3B), the orders exhibited coupled dynamics, demonstrating how perturbations in the Symbolic propagate through prediction errors to the Real and Imaginary. Higher precision values (2.0, Figure 3C) intensified this inter-order coupling, resulting in more extensive system-wide propagation of the initial perturbation. Additionally, we examined configurations with different weight values across connections (Figure 3D), revealing diverse dynamic patterns in the system's behavior.

Our FEP-RSI model formally operationalizes Lacan's fundamental RSI classification system. The neuropsychoanalytic mapping (Table 1) provides the conceptual foundation, while the message-passing mechanism implements the interdependent linkages characteristic of the Borromean knot (Dall'Aglio, 2019)—a structure that parallels the intrinsic connectivity of the brain (Park and Friston, 2013). Significantly, Lacan's assertion that the RSI Borromean knot is sustained by object petit a finds direct correspondence in our model, wherein object petit a functions as the divergence between the internal model and the external environment. This divergence manifests as prediction errors (quantified by variational free energy) that drive the message-passing between orders. This section demonstrates how Lacan's RSI classification system can be formalized within the framework of the interconnected predictive brain in cognitive science.

### 4.2 Dyadic synchronization: desire as generalized synchronization

Extending our individual FEP-RSI model, we investigated desire as a mechanism of generalized synchronization between two subjects. Lacanian psychoanalysis conceptualizes desire, in a linguistic fashion, as a *metaphor* in the Symbolic order (Fink, 2017). In Lacanian terms, a metaphor represents an operation wherein one signifier substitutes for another by appropriating its signified. For example, in the metaphor “John is a lion,” the signifier John appropriates the signified (courage) of the signifier lion. This linguistic structure provides the foundation for Lacanian interpretation of desire: Lacan positions the subject as a signifier within the Other (the *treasure-trove of signifiers*), where desire manifests as the subject's attempt to occupy the position of signified in relation to another signifier (the desired object) (Sheikh, 2017). When one subject desires another, they essentially attempt to position themselves as the signified within the other's Symbolic order—seeking to complete their own lack through the other's attributes, such as success or social recognition.

Within the FEP framework, interpersonal dynamics emerge through mutual predictive inference between agents. This process occurs when two internal models become functionally coupled through reciprocal inference, causing their respective states to exhibit *generalized synchronization* (Friston and Frith, 2015). This synchronization process structurally parallels the Lacanian metaphoric operation: two internal models (analogous to signifiers) converge toward inferring the same hidden state (analogous to the signified). Consequently, desire, in Lacanian terms, can be interpreted as an attempt at generalized synchronization between the Symbolic orders of interacting subjects.

**Figure 4:**
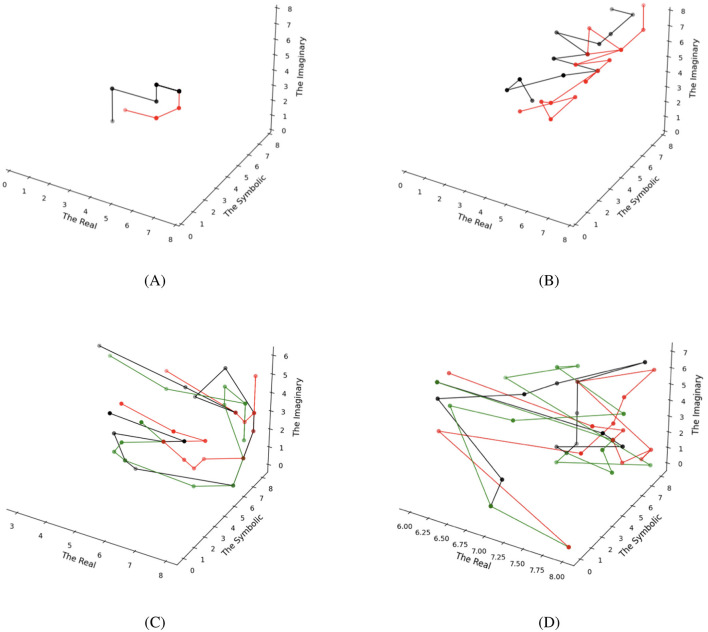
Multi-agent simulations capturing desire (dyadic) and the emergent Other (triadic). **(A, B)** illustrate desire as generalized synchronization between two subjects, with black and red lines. **(C, D)** extend the model to three agents (black, red, and green lines) in a triangular desire configuration, giving rise to collective dynamics interpreted as the Other.

To formalize desire as generalized synchronization, we implemented a dyadic simulation in which two agents, each equipped with identical FEP-RSI internal models but initialized with different states, interact through their respective Symbolic orders. Following Lacan's conceptualization of desire as metaphor, each agent infers the other's Symbolic state as its own preference, thereby operationalizing desire as an attempt to synchronize internal models. Figures 4A, B illustrates the resulting synchronization dynamics over 15 time steps. Despite identical parameters, the system exhibits diverse synchronization patterns in subsequent dynamics. Figure 4A shows a complete convergence between subjects' internal models across all three orders. It echoes what Lacan describes as the mythic state of fusion between infant and mother, an idealized end-point of desire (De Kesel, 2009). Our simulation provides a computational account of how such a hypothetical state of complete intersubjective fusion might emerge within a highly simplified model of dyadic interaction. In contrast, Figure 4B shows “partial” synchronization in the Symbolic orders, while the Real and Imaginary orders maintain relative independence. This pattern demonstrates how synchronization initiated in the Symbolic order can propagate indirectly to influence the other two orders through their Borromean interconnections. These findings align with empirical studies in interpersonal psychology documenting how romantic partners develop shared physiological responses, emotional regulation patterns, and representational systems across multiple temporal scales (Butler and Randall, 2013; Ogolsky et al., 2022; Palumbo et al., 2017).

By extending our FEP-RSI framework to interpersonal dynamics, in which subjects achieve generalized synchronization primarily through their Symbolic orders, we have developed a formal computational operationalization of Lacan's metaphoric structure of *desire*. Furthermore, this synchronization process can also be viewed as a partial computational interpretation of Lacan's *formula of fantasy*:


S◇a

This formula represents the structure of desire's operation (Lacan, 2011), consisting of three elements: the barred subject (S), the diamond operator (◇), and object petit a (*a*). To simple terms, the barred subject represents the subject's alienation by the Other. The diamond operator indicates the barred subject's continuous attraction to object petit a, the cause of desire. In our simulation, aspects of this structure emerge through the generalized synchronization process, as each subject's internal model becomes partially determined by the other's Symbolic order (alienation), while continuously adjusting its states to minimize free energy in pursuit of the desired object (attraction toward object petit a).

Although this dyadic formalization captures the basic structure of desire, a comprehensive account of Lacanian desire necessitates the incorporation of the Other. The following section extends our model to three-agent dynamics to formalize how desire is fundamentally structured by the Other's desire.

### 4.3 Triadic emergence: the Other as collective desire behaviors

Building on our individual and dyadic FEP-RSI models, we develop a triadic model to formalize another key concept—the Other. This extension aligns with recent applications of FEP to collective behaviors and cumulative cultural evolution (Kastel and Hesp, 2021; Kastel et al., 2023). When each participant operates on the principle of minimizing free energy, diverse collective dynamics can emerge spontaneously without explicitly setting any rules (Heins et al., 2024; Ramstead et al., 2021). This emergent property provides a natural bridge to the concept of the Other, which manifests in language, social norms, and culture (Evans, 2006). Consistently, Dall'Aglio (2024) proposed that shared generative models and federated inference can serve as a framework for conceptualizing the Lacanian Other.

In our simulation, we implemented three agents with identical FEP-RSI models arranged in a triangular desire structure, where A desires B, B desires C, and C desires A. The results reveal distinct patterns of group dynamics' simple patterns in Figure 4C and complex patterns in Figure 4D. This simulation offers a computational handle on the concept of the Other, which resides in the Symbolic order at the societal level. As each individual minimizes its free energy within interpersonal desire dynamics, the triad as a whole generates rich collective behaviors. This emergent phenomenon suggests that the Other can be conceptualized as the collective dynamics of human society functioning as a self-organized complex system (Sawyer, 2005).

The triangular desire structure was designed to investigate the relationship between an individual's desire and the Other. Within this structure, each subject desires an object who desires another object, creating a transformation of desires: A's desire becomes oriented not simply toward B, but toward becoming the object of B's desire, which is directed toward C. This process transforms the original desire into a desire to be desired. It thus becomes “the desire of the other.” This transformation aligns with the Hegelian interpretation of desire, upon which Lacan developed his theory (Butler, 1987). Our simulations demonstrate that this configuration generates collective dynamics that constrain each participant's desire. Consequently, the original desire is firstly transformed into the desire of the specific other (in Hegelian theory), and ultimately evolves into the desire shaped by the collective dynamics, i.e., the Other (in Lacanian theory). Interestingly, this finding provides a computational reproduction of how Lacan develop the assertion that “man's desire is the desire of the Other” from Hegelian interpretation that “man's desire is the desire of the other” (Lacan, 2001).

Our simulations reveal that the emergent collective dynamics, while irreducible to individual components, exhibit inherent sensitivity and non-determinism. This computational observation demonstrates that the Other itself is unstable and incomplet—in Lacan's words, “the Other does not exist” (Lacan, 2001). This essential incompleteness of the Other precludes the possibility of achieving wholeness for the subject. It thereby creates the primary condition of subjectivity in Lacanian psychoanalysis: an endless pursuit of fulfillment that is structurally impossible to attain.

## 5 Discussion

This study began by exploring the theoretical compatibility between Lacanian psychoanalysis and the free energy principle (FEP). Fundamentally, both frameworks share a Kantian epistemological premise of the “inaccessibility” of the external world, distinguishing between external reality and a subject's internal representations. The subject must rely on an internal representational system to construct its own subjective reality of the external world. In Lacan's theory, the object petit a represents the gap between material reality and psychic reality, driving the subject's desire to address this gap. In the FEP, free energy quantifies the discrepancy between the internal model and the external world, prompting the system to update its model or take action to reduce it. In both theories, these failures of representation motivate the subject to evolve its representational systems. Regarding representational processes, both approaches reveal that subjective time does not unfold linearly forward but is formed by a dynamic process of forward prediction and backward revision intertwined over time. In summary, Lacanian psychoanalysis and the FEP exhibit a high degree of congruence in their epistemological grounding, in the constructive nature of their representational systems, in the critical role of representation failures, and in their nonlinear temporal structure. Such convergence provides a basis for incorporating Lacanian concepts into the mathematical framework of the FEP, opening up new interdisciplinary dialogues for understanding how the subject operates across diverse scales. Although we leverage the Kantian epistemological concept of “thing-in-itself” to bridge Lacanian psychoanalysis and the FEP, we recognize that Lacanian theory can be studied from multiple philosophical perspectives, especially Hegelian interpretations (Zizek, 2014). Exploring such Hegelian viewpoints might offer complementary insights to an FEP-based understanding of Lacanian psychoanalysis (Boonstra and Slagter, 2019).

Grounded in this theoretical convergence, we developed a series of computational models aimed at providing a formalization of core concepts in Lacanian psychoanalysis. Drawing on a neuropsychoanalytic mapping of Lacan's three orders (RSI) to distinct brain networks, we developed a computational FEP-RSI model that treats each order—Real, Symbolic, and Imaginary—as an FEP unit. Although our mapping is not intended as a strict anatomical delineation, it provides an intuitive way to ground Lacan's abstract RSI framework in neural terms. By implementing each order's perception-action cycle under the FEP, we can computationally capture how these orders interconnect through message-passing mechanisms. Our results show that the FEP-RSI model can reproduce key Lacanian ideas. First, the individual-subject simulations exhibit the Borromean knot's interdependence (the topological structure representing the interrelation of RSI in Lacanian theory). Varying the precisions among the Real, Symbolic, and Imaginary reveals how stronger inter-order connections propagate such perturbations more extensively. Interestingly, the inter-order connection strengths offer a computational analog to Lacanian conceptualizations of varying RSI organizational structures and related symptoms. For instance, psychosis has been theorized as a failure in the “slippage” of RSI (Mills and Downing, 2018), which demonstrates the altered precision weights across different orders. Within our FEP-RSI model, this can be translated as the “disconnection hypothesis” of schizophrenia as well, where aberrant connectivity between brain regions is considered a core pathophysiological mechanism (Friston et al., 2016). Further research, potentially incorporating empirical data or more detailed clinical phenomenology, would be necessary to validate and explore how precisions within an FEP-RSI framework could account for the diverse structural organizations and disturbances in psychopathology.

From an FEP perspective, interpersonal desire, a metaphorical process, can be viewed as the attempt to achieve generalized synchronization in the Symbolic order of two agents. In our simulations, two subjects with identical FEP-RSI models but different initial states continuously inferred each other's Symbolic states. This simplified scenario illustrates the ultimate goal of desire: a unified state with the object of desire achieved through complete synchronization. Such generalized synchronization, through minimizing free energy, effectively enacts Lacan's notion of desire as the subject's continual drive to reduce the gap represented by object petit a. While our model captures the functional dynamics of alienation and attraction within the formula of fantasy, we acknowledge that *fantasy* itself encompasses far more complex and nuanced meaning within Lacanian theory. These include its role in separation from the Other, its function as an axiom structuring the subject's reality, and its complex topological properties beyond what our current implementation addresses (Lacan, 2011). Moreover, our formalization of desire primarily captures its metaphoric dimension (the attempt to occupy the Other's position) while not yet modeling its equally crucial metonymic aspect. In Lacanian theory, desire operates through *metonymy* longitudinally, continuously sliding from one object to another, leading to an endless chain of desire (Sheikh, 2017). A more complete formalization would need to implement both mechanisms: the metaphoric (synchronic identification with the Other) and the metonymic (diachronic displacement between objects over time).

Expanding from individual and dyadic FEP-RSI models to a triadic scenario, we used three interacting agents to illuminate Lacan's crucial concept of the Other as a collective dynamic of society. Each subject's pursuit of an object that desires a different object binds them into a collective process where emergent patterns unfold. The collective dynamics, in turn, constrain each participant's desire, exemplifying Lacan's dictum that “man's desire is the desire of the Other.” The simulation also reveals that the Other is neither a static nor deterministic entity, supporting Lacan's claim that “the Other does not exist.”

In summary, our study presents a novel demonstration that the free energy principle can be harnessed to formalize Lacanian psychoanalysis, establishing a computational framework that bridges these two domains. While our study offers a preliminary exploration, we acknowledge the limitations of the current approach. This framework remains highly abstract and represents a simplified interpretation of Lacan's complex theoretical system. Future studies could extend this model by incorporating continuous state spaces, hierarchical network architectures, more refined cognitive modules, and simulations of multi-agent interactions within realistic social contexts.

## Data Availability

The original contributions presented in the study are included in the article/supplementary material, further inquiries can be directed to the corresponding author.

## Footnotes

^1^ https://github.com/DigitalTwinMind/ActiveInferenceLacan
